# Environmental Drivers of Trace Element Variability in *Hypnum cupressiforme* Hedw.: A Cross-Regional Moss Biomonitoring Study in Georgia and the Republic of Moldova

**DOI:** 10.3390/plants14132040

**Published:** 2025-07-03

**Authors:** Omari Chaligava, Inga Zinicovscaia, Liliana Cepoi

**Affiliations:** 1 Joint Institute for Nuclear Research, 6 Joliot-Curie Str., 141980 Dubna, Russia; zinikovskaia@mail.ru; 2 Doctoral School of Natural Sciences, Moldova State University, 75A M. Kogalniceanu Str., MD-2009 Chisinau, Moldova; liliana.cepoi@imb.utm.md; 3 Andronikashvili Institute of Physics, I. Javakhishvili Tbilisi State University, 6 Tamarashvili Str., 0162 Tbilisi, Georgia; 4 Horia Hulubei National Institute for R&D in Physics and Nuclear Engineering, 30 Reactorului Str., 077125 Magurele, Romania; 5 Institute of Microbiology and Biotechnology, Technical University of Moldova, 1 Academiei Str., MD-2028 Chisinau, Moldova

**Keywords:** moss biomonitoring, *Hypnum cupressiforme* Hedw., trace elements, atmospheric deposition, environmental variables, Georgia, Moldova

## Abstract

This study investigates the influence of environmental variables on the elemental composition of *Hypnum cupressiforme* Hedw. mosses in Georgia and the Republic of Moldova, within moss biomonitoring studies aimed at analyzing atmospheric deposition patterns. Moss samples of *Hypnum cupressiforme*, characterized by a cosmopolitan distribution and a wide range of habitats, were collected from diverse geographical and climatic zones and analyzed for Al, Ba, Cd, Co, Cr, Cu, Fe, Mn, Ni, Pb, Sr, V, and Zn. Statistical methods (Spearman correlations, PCA, Kruskal–Wallis tests) were applied to evaluate interactions between elemental concentrations and factors such as topography, climate, land cover, etc. Results revealed strong correlations among lithogenic elements (Al, Co, Cr, Fe, Ni, and V), indicating natural weathering sources, while Cu exhibited potential anthropogenic origins in the Republic of Moldova. Elevated Cd and Pb levels in Georgian high-altitude regions were linked to wet deposition and steep slopes, whereas Moldovan samples showed higher Sr and Zn concentrations, likely driven by soil erosion in carbonate chernozems. The study highlights geogenic and climatic influences on element accumulation by moss, offering insights into the effectiveness of moss biomonitoring across heterogeneous landscapes.

## 1. Introduction

Monitoring pollution using moss bioindicators is a cost-efficient and practical tool, offering broad spatial coverage for tracking contaminants such as trace metals, nitrogen, organic pollutants, and microplastics [1,2,3]. Unlike traditional approaches that rely on costly, fixed monitoring stations, this method, requiring minimal technical expertise or specialized equipment, leverages the structural and functional features of naturally growing moss species. These organisms are widespread, with remarkable ecological plasticity. They thrive on a wide range of substrates, including natural surfaces (tree bark (both coniferous and deciduous), rocks (especially acidic substrates, e.g., sandstone, granite), soil, and decaying wood), and human-altered environments (walls, fences, and even urban structures). The key adaptation of mosses is their high surface-to-volume ratio, with nutrient uptake happening predominantly from the atmosphere. The ability to capture and retain airborne particles reflects localized deposition trends, enabling moss tissues to record temporal changes and act as biological filters, integrating long-term environmental exposure to pollutants [1,4,5]. While moss analysis does not yield direct measurements of atmospheric deposition rates, it enables qualitative and semi-quantitative assessments of pollution through advanced data interpretation [6,7]. This methodology can compensate for the lack of information in environmental monitoring systems, especially in areas that are remote and under-researched on a global scale, where conventional infrastructure is too expensive [8,9].

The elemental composition of mosses is influenced by numerous interacting variables [10,11]. Environmental parameters such as precipitation, wind dynamics, and moisture levels affect elements deposition and retention on moss surfaces [12,13]. Potentially toxic elements (e.g., Pb, Cd, Zn) bind strongly due to their high affinity for cation exchange sites. Long exposure increases accumulation but may lead to saturation of binding sites or displacement of previously adsorbed elements, with lighter elements (e.g., Na, K, P) being more prone to leaching [14,15]. Mosses exhibit higher accumulation of certain elements due to increased solubility, as dissolved elements are adsorbed directly to the surface layers of the moss [16]. Research by Wang et al. [17] highlighted the role of mosses in capturing atmospheric depositions, particularly in alpine regions with high precipitation, where wet deposition enhances the uptake of soluble elements like Cd and Pb. Mosses serve as effective indicators of pollution hotspots, including industrial zones and high-traffic roads [18,19,20]. The study by M. Aničić Urošević et al. [21] demonstrated that mosses, particularly *Hypnum cupressiforme*, could identify rare earth element (REE) anomalies caused by industrial pollution. Species-specific traits in mosses, including structural and physiological differences, also influence their ability to absorb and retain elements [13,22]. Furthermore, soil characteristics and regional geology may introduce elements via windblown dust from the surrounding terrain [21]. Broader atmospheric processes, such as the cross-border transport of pollutants, additionally lead to differences in elemental profiles [23,24,25,26,27]. Accounting for these multifaceted influences is critical to ensuring reliable analysis of biomonitoring results and effective assessment of ecosystem contamination.

Georgia, as well as the Republic of Moldova, participates in the moss biomonitoring program of UNECE ICP Vegetation since 2014–2015 [28,29]. The 2015–2016 Moss Survey report revealed that the elemental content in both countries was notably higher compared to other nations participating in the program [30].

In their 2023 article, Chaudhuri et al. [1] highlighted the potential of mosses as complementary tools for ambient air quality monitoring, particularly in regions where traditional sensor-based networks are resource-intensive to deploy, but the authors emphasize that further research is required to assess the potential effects of localized natural factors, such as pH fluctuations, weather patterns, and terrain features, on the bioaccumulation of pollutants. Studies like Oishi [31] demonstrated that natural factors such as slope aspect, altitude, and microclimatic conditions (e.g., pH, humidity) significantly influence bioaccumulation patterns in mosses (e.g., nitrogen levels in *Hylocomium splendens*). Thus, these variables must be systematically studied to improve the interpretability of moss-based data.

By integrating data from moss surveys of Georgia and Moldova, the research aims to elucidate how variables such as topography, land cover, elevation gradients, and climatic conditions shape the chemical composition of moss tissues. This approach provides valuable insights into the effectiveness of biomonitoring methods across diverse geographical settings, particularly in areas characterized by varying climate patterns and landscape features.

## 2. Results and Discussion

The summary statistics for 13 elements and sampling site characteristics are shown in Table 1, including both the initial dataset and the adjusted dataset, modified by Grubbs’ test for outlier removal to eliminate local anomalies.

**Figure 1:**
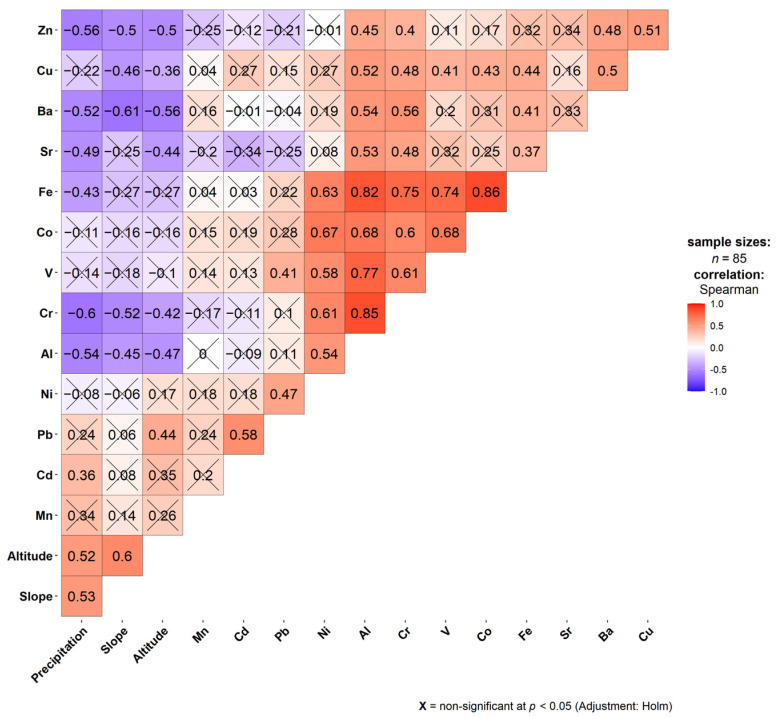
Correlation matrix between the elements of the entire adjusted dataset. X stands for not significant.

The correlation matrix of the adjusted dataset showed a strong positive correlation among Al, Co, Cr, Fe, Ni, and V, with correlation coefficient (r) values ranging from 0.54 to 0.86 (Figure 1). These strong correlations suggest a shared origin for these elements, most likely derived from natural sources such as crustal materials or soil dust [32]. On the other hand, additional factors like average precipitation, slope degree, and altitude showed significant negative correlations (r= −0.42 to −0.61) with elements such as Zn, Ba, Cr, Al, as well as Cu (in the case of slope), Fe (for precipitation) and Sr (for precipitation and altitude). Moreover, these environmental variables (precipitation, slope, and altitude) exhibited strong correlations with each other, indicating interrelated influences on elemental distribution.

**Figure 2:**
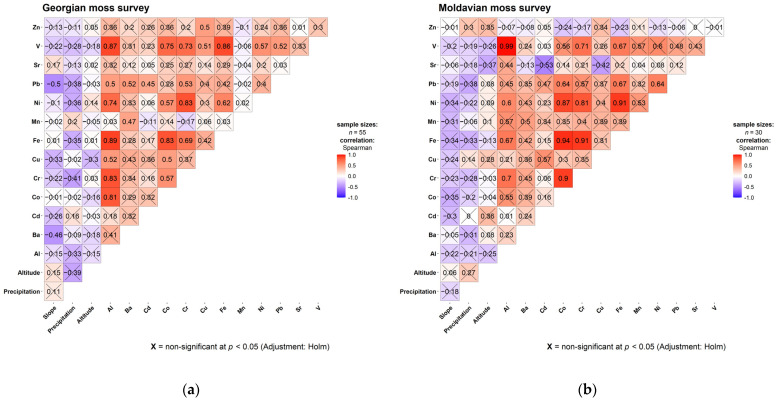
Correlation matrices of Georgian (**a**) and Moldovan (**b**) moss surveys’ adjusted dataset. Non-significant correlations are marked with X.

The correlation matrices for both the Georgian and Moldovan moss survey data (Figure 2) also revealed a strong positive correlation among Al, Co, Cr, Fe, Ni, and V. The correlations between Al and lithogenic elements were stronger in the Georgia survey compared to the Moldova survey, while the opposite pattern was observed for Fe. A notable exception was the V-Al correlation in the Republic of Moldova, which was exceptionally high (r = 0.99); other notably high correlations included Cr-Co (r = 0.90), Fe-Co (r = 0.94), Fe-Cr (r = 0.91), Fe-Ni (r = 0.91), Co-Ni (0.87), and Ni-Cr (0.81). For environmental variables, a significant negative correlation was observed only in Georgia—specifically between slope degree and Ba (r = −0.46) and between slope degree and Pb (r = −0.50).

**Figure 3:**
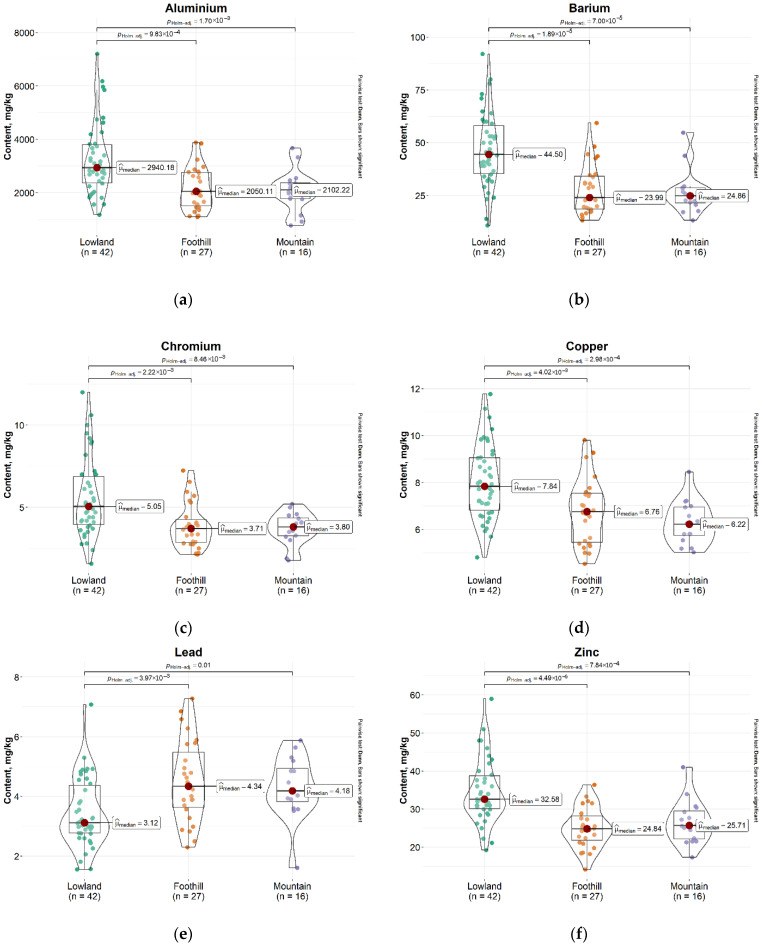

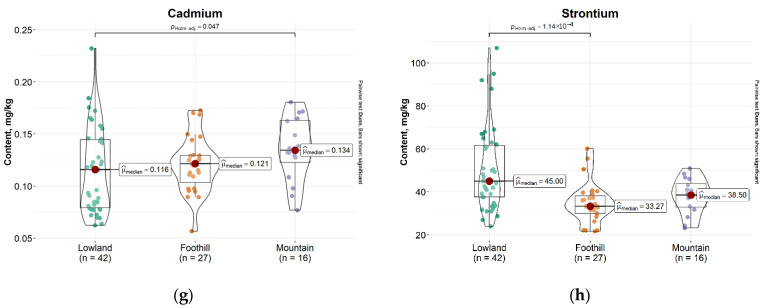
Boxplots showing elemental content in mosses across three elevation zones (Lowland, Foothill, Mountain) for: (**a**) Aluminium; (**b**) Barium; (**c**) Chromium; (**d**) Copper; (**e**) Lead; (**f**) Zinc; (**g**) Cadmium; (**h**) Strontium. Significant pairwise differences (Kruskal–Wallis with Dunn’s post hoc, *p* < 0.05) are indicated with connecting lines.

When comparing all data across different altitude zones (lowland, foothill, and mountain areas), the Kruskal–Wallis test revealed significant differences in the content of several elements. Specifically, Al, Ba, Cr, Cu, Pb, and Zn showed significant variations between lowland and foothill zones, as well as between lowland and mountain zones. In the case of Cd and Sr, significant differences were observed only between lowland and mountain areas. The median concentrations were generally higher in lowland areas for most elements; however, Pb and Cd exhibited higher values in foothill and mountain zones. These results are visually summarized in Figure 3.

The climate zone-based data comparison showed significant differences for Ba between Dfa and Dfb, for Cd between BSk and Cfa, and for Pb, Sr, and Zn between Cfa and Dfa (Figure S1). It should be noted that Dfa (cold, no dry season, hot summer) and BSk (cold arid steppe) are mostly associated with Moldovan samples, while Dfb (cold, no dry season, warm summer) and Cfa (temperate, no dry season, hot summer) with Georgian ones. Additionally, sites in Dfa and BSk typically experience considerably lower annual precipitation compared to those in Dfb and Cfa.

The samples associated with BSk sites exhibited the highest median content of Al, Co, Cr, Fe, Sr, and Zn, along with the lowest levels of Cd and Pb. These observations suggest a potential influence of wind erosion and local geological composition, while low rainfall might contribute to the reduced Cd and Pb content [33]. However, these interpretations remain hypothetical at this stage, due to the small number of samples from the BSk climate zone, and further sampling is required to confirm these trends and clarify potential environmental or geochemical drivers.

A comparison of similar parameters—such as Dfb climate, mixed forest cover, population density, slope terrain, and lowland zones—between countries revealed no significant differences. However, Moldovan samples exhibited higher median content of Al, Ba, Cr, Sr, and Zn, whereas Georgian samples had elevated levels of Cd, Mn, and Pb.

**Figure 4:**
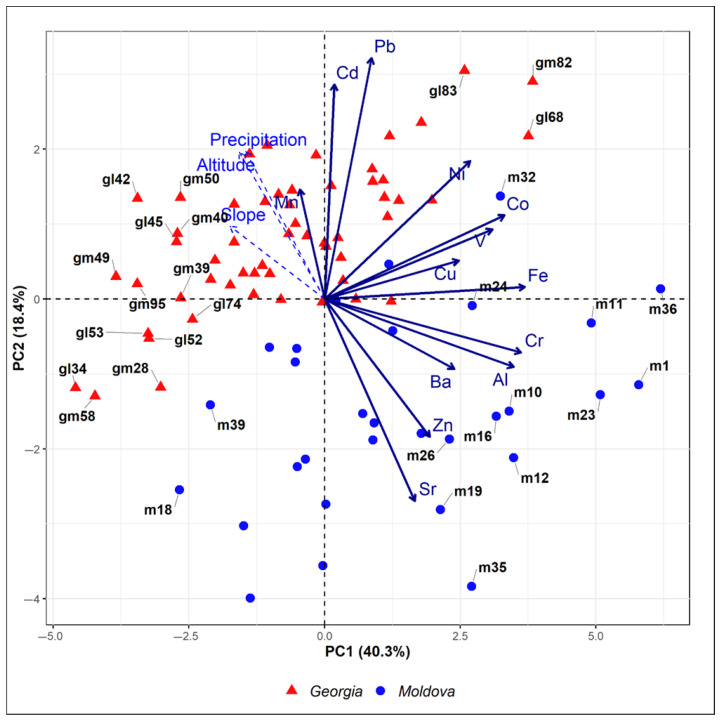
The biplot of the first two principal components expresses 58.7% of the variance in the data. Samples are marked with a unique combination of color and symbol, dependent on the country. Variables appear as arrows from the origin, with qualitative variables shown as dashed arrows.

**Figure 5:**
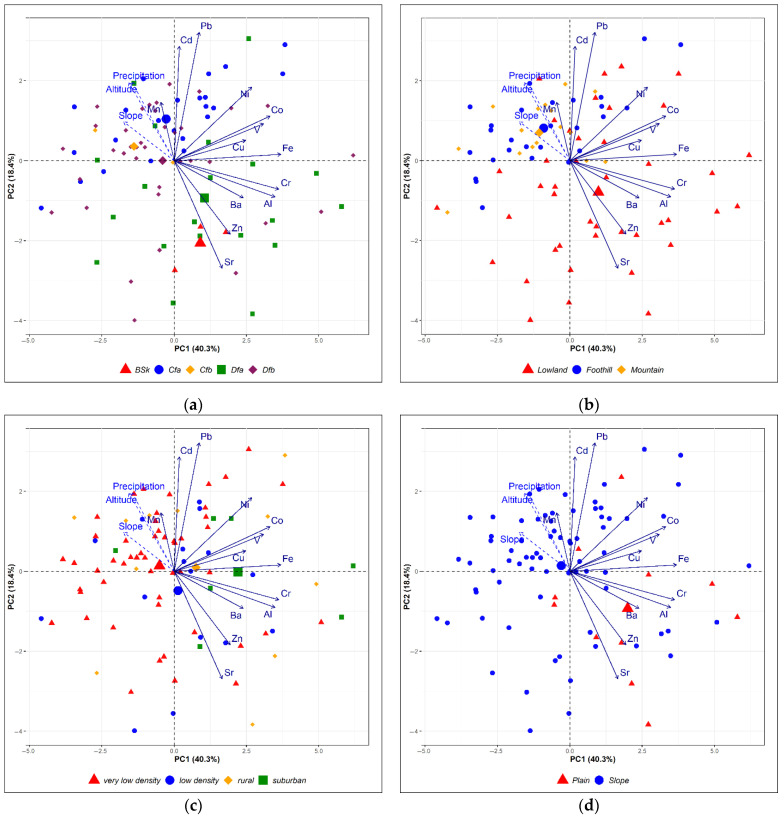
Biplots of the first two PCs. Samples are marked with a unique combination of color and symbol, dependent on the climate (**a**), elevation zone (**b**), population density (**c**), and terrain (**d**).

The results of the principal component analysis (PCA) are presented in Figure 4 and Figure 5, displaying the first two principal components, which together explain 58.7% of the total variance (PC1: 40.3%; PC2: 18.4%). The biplot illustrates distinct patterns of variable contributions, with Fe, Cr, Al, Co, V, Ni, Cu, and Ba showing the strongest influence on PC1, while PC2 was predominantly shaped by Pb, Cd, Sr, and Zn.

The strong association of Fe, Cr, Al, Co, V, Ni, and Ba with PC1 suggests these elements originate from natural weathering processes dominant in the Moldova samples, driven by extensive soil erosion and suspension under sparse plant coverage and dry climatic conditions [32,34]. Additionally, agricultural practices likely contribute to their release and mobilization. In the case of Georgia, it was also observed that agricultural activities—especially during dry seasons—enhance soil particle emissions, while open landscapes aid their widespread dispersal [35]. However, in this study, due to the limited number of moss samples collected near agricultural lands, most were excluded during outlier testing.

Copper shows a weaker loading on PC1 compared to other elements, indicating it is less strongly tied to the geogenic signal. This deviation may reflect anthropogenic inputs, as Cu is widely used in agrochemicals as fertilizers and fungicides in agricultural areas of Moldova [36].

In the biplot, the supplementary variables altitude, slope degree, and precipitation (projected post hoc onto the PCA space) cluster closely along the positive direction of PC2, and since Pb and Cd also show positive correlations with PC2, this suggests that higher elevations, steeper slopes, and increased precipitation are associated with greater accumulation of these metals in mosses. This aligns with Wang et al. [17] who showed that mosses in alpine regions with high precipitation effectively capture soluble elements like Cd and Pb through wet deposition. Additionally, Aboal et al. [33] observed strong correlations between Cd/Pb levels in mosses and bulk deposition, reinforcing that these metals are reliably tracked by moss biomonitoring in such environments. The clustering implies that topographic and climatic factors (e.g., altitude-driven precipitation) may enhance atmospheric deposition and pollutants uptake by moss.

Based on the Wilks test *p*-value, the qualitative variables that best explain the distances between individuals in the PC1-PC2 biplot, ranked in descending order of importance, are country, elevation zones, land cover, climate, and terrain (Figure 5).

In PCA, the group containing individuals m8, m19, m13, m34, m41, and m35 (which have negative coordinates on the axis) can be characterized by high values for the variables Sr and Zn, and low values for the variables Cd, Pb, altitude, precipitation, and slope.

According to Kabata-Pendias et al. [37], the highest Sr concentrations are found in heavy loamy soils, especially developed in hot and arid climates. In the Republic of Moldova, soil erosion poses a significant environmental challenge, especially on sloping terrains and in areas with fragmented relief, as highlighted by Bejan et al. [34]. Among the most impacted soil subtypes are carbonate chernozems, which typically have a loamy texture and are predominantly found in the southeastern and southern regions of the country—areas characterized by a BSk climate type (Figure 7). Given these findings, it is reasonable to conclude that soil erosion is the primary contributor to elevated Sr levels in moss samples of Moldova. Zinc concentrations in Moldova also appear to be influenced by soil composition, as Moldovan soils exhibit higher Zn levels compared to those in Georgia [38,39].

## 3. Materials and Methods

### 3.1. Study Area

**Figure 6:**
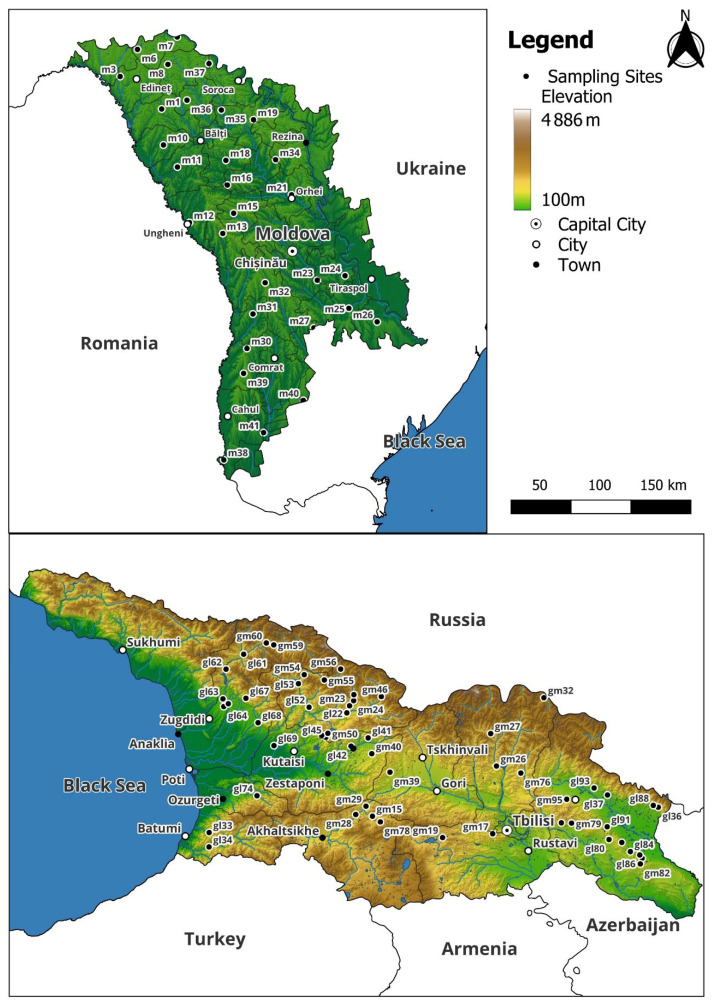
Moss sampling maps for the Republic of Moldova and Georgia.

Both countries are situated within the Black Sea basin, sharing proximity to its climatic influences, yet they exhibit notable differences in relief. While the Republic of Moldova is characterized by relatively lowland and hilly terrain, with over 80% of its terrain below 200 m elevation, Georgia is predominantly mountainous, with approximately two-thirds of its terrain at high elevations and roughly 20% lying above 2000 m or more above sea level (Figure 6).

**Figure 7:**
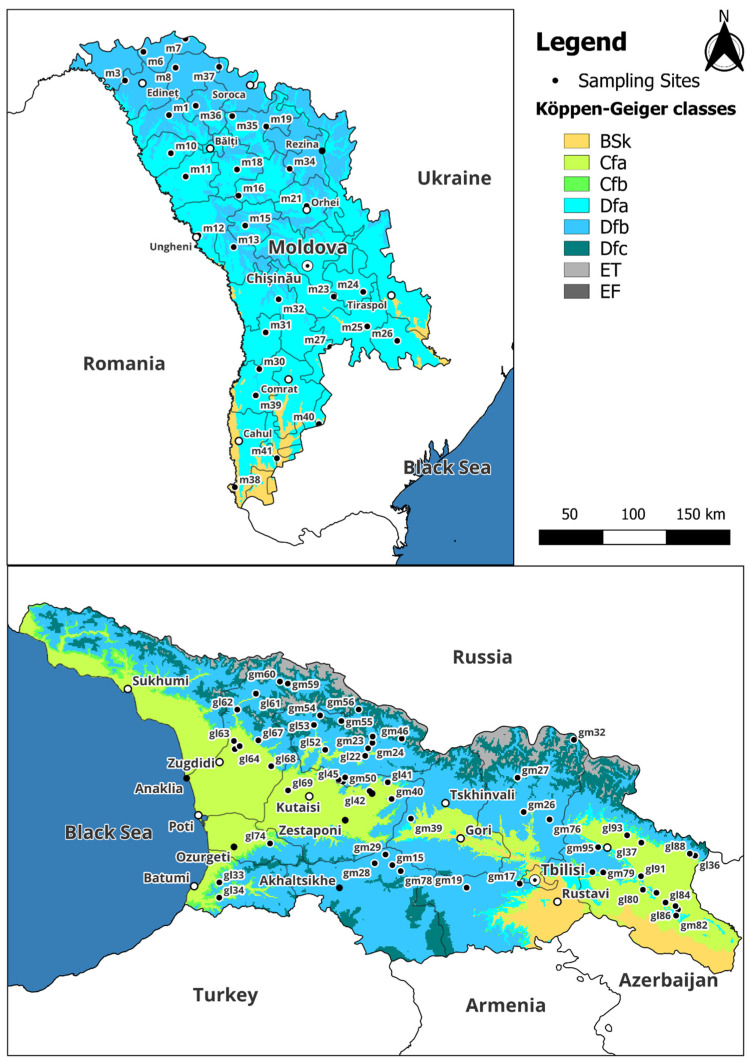
Köppen–Geiger classification for the sampling sites. (BSk—Arid, steppe, cold; Cfa—Temperate, no dry season, hot summer; Cfb—Temperate, no dry season, warm summer; Dfa—Cold, no dry season, hot summer; Dfb—Cold, no dry season, warm summer; Dfc—Cold, no dry season, cold summer; ET—Polar, tundra; EF—Polar, frost [24]).

The Republic of Moldova is a predominantly rural country; 74% of its total area is agricultural land, whereas in Georgia, the same parameter is only 43%. The main economic activities also differ in many ways. The economy of Moldova remains heavily agrarian, focusing on cereals, sunflowers, and viticulture. Georgia, while also renowned for its wines, leverages a more diversified economy, including tourism, mining, and hydropower, capitalizing on its mountainous resources and Black Sea coastline [40,41]. Despite these contrasts, they encompass overlapping climate zones, as classified by the Köppen–Geiger classification (Figure 7), and the same species of mosses are available on both territories, providing a valuable basis for analyzing the environmental factors that contribute to elemental accumulation in mosses.

### 3.2. Moss Sampling

In April 2020, a moss survey was conducted in Moldova, while in Georgia, to achieve nationwide coverage, the moss survey was carried out in phases from 2019 to 2023, with fieldwork conducted at the end of spring and summer months. Moss samples were collected following the ICP Vegetation moss survey protocol [31]. Samples were stored in air-permeable bags, and disposable polyethylene gloves were used to avoid contamination. Moss shoots were cleaned of extraneous materials with plastic tweezers after all samples were dried at 105 °C for 48 h until reaching a constant weight.

### 3.3. Sample Preparation and Analysis

Moss samples from Moldova were analyzed using instrumental neutron activation analysis (INAA) at the REGATA experimental installation of the IBR-2 reactor (JINR, Dubna, Russia). Additionally, atomic absorption spectroscopy (AAS) was performed using a Thermo Fisher Scientific (Waltham, MA, USA) iCE 3400 AAS equipped with electrother-mal atomization. Moss samples from Georgia were analyzed via inductively coupled plasma optical emission spectrometry (ICP-OES) using an Analitik Jena (Jena, Germany) PlasmaQuant PQ 9000 Elite ICP-OES spectrometer.

Sample preparation for AAS and ICP-OES followed similar protocols, involving drying, microwave-assisted acid digestion (HNO_3_ + H_2_O_2_), and dilution to 50 mL with distilled water. In case of INAA, dried moss was pressed into pellets and packed in polyethylene bags (for short-lived isotopes) and aluminum cups (for long-lived isotopes).

Both studies ensured low contamination risks and followed strict protocols for reliable results. Quality control for ICP-OES, NAA, and AAS analytical methods was performed using certified reference materials (CRMs) (Table S1). For ICP-OES, recovery rates for elements measured in CRMs M2 (Pleurozium schreberi), INCT-PVTL-6 (Polish Virginia Tobacco Leaves), and INCT-OBTL-5 (Oriental Basma Tobacco Leaves) fell within acceptable ranges. NAA quality control used NIST SRM 1547 (Peach Leaves), NIST SRM 2710 (San Joaquin Soil), NIST SRM 2709a (San Joaquin Soil), NIST SRM 1632c (Bituminous Coal), and INCT-OBTL-5. AAS validation relied on NIST SRM 1575a (Trace Elements in Pine Needles) and INCT-OBTL-5. The shared use of INCT-OBTL-5 across ICP-OES, NAA, and AAS ensured consistent accuracy validation for all methods. The sample preparation, analysis procedure, and quality control results are described in detail in previous works [32,35,36].

### 3.4. Study Design and Data Collection

In order to gain deeper insight into the factors influencing elemental content in mosses, a comparative study was conducted combining two moss surveys from distinct regions: the Republic of Moldova and Georgia [32,35]. A single moss species was selected to minimize the confounding effects of interspecies variability on element accumulation patterns. This critical factor, highlighted by Schröder et al. [22], demonstrated significant species-specific differences in Cd, Cu, Ni, Pb, Zn, N, and Hg content, aligning with their prior multivariate studies. Our study focused on *Hypnum cupressiforme* Hedw., a widely distributed and ecologically resilient moss species frequently used in biomonitoring [27,42,43,44]. Crucially, this species was the only candidate that was collected across both surveyed countries, enabling comparison while eliminating taxonomic bias. To isolate the effects of natural environmental factors and minimize distortion from samples associated with direct human activity and/or local hotspots, the Grubbs’ outlier test was used to exclude data.

To enhance the characterization of the sampling location, the high-resolution Köppen–Geiger climate classification map for the period 1991–2020 was utilized [45]. This decision was based on the exceptional accuracy of the map, derived from the integration of advanced statistical methods and modern climatological datasets. The spatial resolution of approximately 1 km^2^ enables precise delineation of microclimatic zones, ensuring that local environmental conditions at the sampling site are captured with minimal spatial uncertainty. Furthermore, the temporal coverage (1991–2020) aligns with the World Meteorological Organization standard 30-year reference period for climate normals [46], thereby providing a robust representation of recent climatic trends. The map’s scientific rigor, including validation through peer-reviewed methodologies and cross-referencing with ground-based observational data, reinforces its reliability for ecological and environmental studies. Using this resource, regional climate variability was systematically contextualized, facilitating replicable comparisons with other global studies adhering to the Köppen–Geiger model.

In terms of population density, each sampling point was characterized by aggregating all 1 km resolution grid cells from the GHS Settlement Model (GHS-SMOD) within a 2 km radius around it. The grid cell values are derived from the degree of urbanization methodology, where each cell is classified into settlement typologies. The density scale is defined as follows: very low density (Class 11: Very Low Density Rural grid cells, <50 inhabitants/km^2^), low density (Class 12: Low Density Rural grid cells, 50–300 inhabitants/km^2^), rural (Class 13: Rural Cluster grid cells, ≥300 inhabitants/km^2^ in clustered rural settlements), suburban (Class 21: Suburban or Peri-Urban grid cells, transitional zones with moderate density), and urban (Classes 22, 23, 30: Semi-dense/Dense Urban Clusters or Urban Centers, ≥1500 inhabitants/km^2^). The highest observed class value within the 2 km radius around the sampling point was identified to determine the dominant density category, prioritizing urban classes over lower-density classifications [47].

The sampling location landscape was evaluated based on slope degree parameters to classify the terrain. The areas with a slope angle not exceeding 3 degrees were identified as plains, while steeper inclinations were categorized as slopes [48]. High-resolution data was utilized to accurately measure these slope gradients, ensuring precise terrain classification [49,50]. In addition to slope analysis, the altitude of each sampling site was measured using GPS to further characterize the landscape. Elevation data allowed for the classification of sampling locations into distinct altitudinal zones: lowland (0–500 m above sea level), foothill (500–1000 m), and mountain (1000–2500 m). This zonation system reflects elevation-dependent ecological and climatic variations, with lowlands typically associated with warmer, flatter terrains, foothills marking transitional ecosystems, mountains hosting mixed vegetation, as well as cooler, high-elevation habitats.

To estimate precipitation at the sampling point, high-resolution geospatial data (3 arc-second resolution) sourced from Woods et al. [49,51] was used. The dataset provides harmonized annual global precipitation estimates, where each grid cell represents the mean annual precipitation. This was derived by summing all monthly precipitation rasters for the base year (matching the year of sampling) and dividing by 12 to obtain the average.

### 3.5. Statistics

To evaluate pairwise relationships between elemental content, nonparametric Spearman’s rank correlation was applied due to the non-normal distribution of the data (assessed via Shapiro–Wilk tests). The analysis was implemented in R (v4.5.0) using the ggstatsplot package (v0.13.1), which generates correlation matrices with significance levels (α = 0.05) [52]. To mitigate the risk of Type I errors arising from multiple hypothesis testing, *p*-values were adjusted using the Holm–Bonferroni method.

Differences in elemental content across different factors were assessed using the Kruskal–Wallis test, a nonparametric alternative to one-way ANOVA suitable for ordinal or non-normal data. Post hoc pairwise comparisons between zones were conducted using Dunn’s test with Holm-adjusted *p*-values. Results were visualized as annotated boxplots also using ggstatsplot, which displays median values, interquartile ranges (IQR), and outliers (defined as values exceeding 1.5 × IQR) [52].

Principal Component Analysis (PCA) was performed to reduce dimensionality and identify latent patterns in the elemental concentration dataset. Prior to analysis, the data was centered and scaled to unit variance to normalize differences in measurement units. The PCA computation was carried out using the FactoMineR package (v2.11), which allowed for the integration of supplementary data, projected onto the PCA space to evaluate their alignment with elemental patterns without affecting component computation [53]. Visualizations were generated using the factoextra package (v1.0.7) [54].

## 4. Conclusions

The findings highlight significant spatial and climatic variability in elemental accumulation within *Hypnum cupressiforme*, shaped by natural geochemical processes and anthropogenic activities. Georgian samples demonstrated higher Cd and Pb levels, correlating with precipitation and elevation, while Moldova’s lowland regions exhibited elevated Sr and Zn linked to soil erosion. Principal component analysis attributed 58.7% of the variance to lithogenic elements and climatic–topographic variables, reinforcing the role of environmental context in biomonitoring interpretations. Given that elemental content in mosses is influenced by multiple interacting factors, future studies should prioritize collecting larger sample sets with overlapping environmental variables (e.g., shared climatic zones, land cover types, or elevation ranges) to isolate individual drivers and reduce confounding effects. This approach would enhance the interpretability of biomonitoring data and clarify complex interactions between natural and anthropogenic influences. Additionally, expanding spatiotemporal sampling and investigating specific pollution sources, such as agricultural inputs, will refine mitigation strategies in ecologically diverse regions.

## Figures and Tables

**Table 1:** Summary statistics and sample site characteristics from the 2019/2023 moss survey of *Hypnum cupressiforme* in Georgia and the Republic of Moldova, including both initial and adjusted (post-Grubbs’ test) datasets.

Characteristic	Original/Initial Data		Adjusted Data	
	Georgia	Moldova	Georgia	Moldova
	N = 70	N = 41	N = 55	N = 30
**Land Cover** *				
Coniferous forest	4 (5.7%)	0	4 (7.3%)	0
Deciduous forest	38 (54%)	0	28 (51%)	0
Mixed forest	28 (40%)	41 (100%)	23 (42%)	30 (100%)
**Terrain** *				
Plain	6 (8.6%)	19 (46%)	2 (3.6%)	9 (30%)
Slope	64 (91%)	22 (54%)	53 (96%)	21 (70%)
**Köppen–Geiger climate classification** *				
BSk (Arid, steppe, cold)	0	4 (9.8%)	0	3 (10%)
Cfa (Temperate, no dry season, hot summer)	36 (51%)	0	23 (42%)	0
Cfb (Temperate, no dry season, warm summer)	2 (2.9%)	0	2 (3.6%)	0
Dfa (Cold, no dry season, hot summer)	4 (5.7%)	20 (49%)	4 (7.3%)	16 (53%)
Dfb (Cold, no dry season, warm summer)	28 (40%)	17 (41%)	26 (47%)	11 (37%)
**Population density** *				
Very low density	49 (70%)	17 (41%)	38 (69%)	13 (43%)
Low density	10 (14%)	8 (20%)	8 (15%)	8 (27%)
Rural	6 (8.6%)	9 (22%)	6 (11%)	5 (17%)
Suburban	5 (7.1%)	6 (15%)	3 (5.5%)	4 (13%)
Urban	0	1 (2.4%)	0	0
**Elevation zones** *				
Lowland (0–500 m)	20 (29%)	41 (100%)	12 (22%)	30 (100%)
Foothill (500–1000 m)	32 (46%)	0	27 (49%)	0
Low-mountain (1000–1500 m)	14 (20%)	0	12 (22%)	0
Upper-mountain (1500–2500 m)	4 (5.7%)	0	4 (7.3%)	0
**Precipitation** **				
Average Annual Precipitation in mm	848 (629, 1135)	362 (347, 383)	744 (629, 1033)	362 (347, 383)
**Altitude** **				
Meters above mean sea level	749 (442, 1020)	148 (94, 200)	820 (557, 1133)	144(96, 199)
**Topographic slope** **				
Topographic slope in degrees	11 (6, 18)	4 (2, 6)	12 (8, 19)	5 (2, 6)
**Element content in mg/kg** **				
Al	2288 (1660, 2840)	3430 (2680, 4750)	2184 (1636, 2771)	3245 (2680, 4270)
Ba	29 (21, 43)	53 (41, 71)	26 (19, 35)	50 (41, 60)
Cd	0.13 (0.11, 0.17)	0.11 (0.08, 0.15)	0.13 (0.11, 0.15)	0.09 (0.08, 0.12)
Co	0.96 (0.69, 1.19)	0.94 (0.71, 1.28)	0.92 (0.67, 1.12)	0.94 (0.71, 1.28)
Cr	3.88 (2.91, 4.99)	5.40 (4.40, 8.90)	3.80 (2.89, 4.50)	5.35 (4.40, 8.20)
Cu	7.29 (6.16, 8.86)	8.62 (7.14, 9.38)	6.76 (5.64, 7.63)	7.83 (6.70, 9.05)
Fe	1898 (1367, 2436)	2190 (1790, 3100)	1798 (1335, 2208)	2130 (1790, 3070)
Mn	132 (88, 179)	93 (74, 144)	111 (86, 160)	87 (71, 106)
Ni	4.40 (3.52, 5.46)	4.10 (3.30, 5.30)	4.31 (3.42, 5.22)	3.72 (3.24, 4.80)
Pb	4.78 (3.88, 5.92)	3.14 (2.77, 3.70)	4.47 (3.60, 5.19)	3.00 (2.75, 3.29)
Sr	36 (31, 42)	51 (39, 68)	36 (32, 41)	50 (42, 67)
V	5.5 (4.2, 7.4)	5.4 (4.0, 8.0)	5.34 (4.11, 6.86)	5.20 (4.00, 6.90)
Zn	27 (23, 32)	39 (33, 46)	26 (22, 30)	36 (31, 43)

* *n* (%). ** Median (Q1, Q3).

## Data Availability

Data is contained within the article or Supplementary Material.

## Supplementary Materials

The following supporting information can be downloaded at: https://www.mdpi.com/article/10.3390/plants14132040/s1, Figure S1: Boxplots of elemental concentrations across climate zones. Significant pairwise differences (Kruskal–Wallis with Dunn’s post hoc, *p* < 0.05) are indicated with connecting lines; Table S1: Quality control of measurements and LOD values for the determined elements.

## References

[1] Chaudhuri S., Roy M. (2024). Global Ambient Air Quality Monitoring: Can Mosses Help? A Systematic Meta-Analysis of Literature about Passive Moss Biomonitoring. Environ. Dev. Sustain..

[2] Roblin B., Aherne J. (2020). Moss as a Biomonitor for the Atmospheric Deposition of Anthropogenic Microfibres. Sci. Total Environ..

[3] Du C., Guo Q., Zhang J. (2022). A Review on Moss Nitrogen and Isotope Signatures Evidence for Atmospheric Nitrogen Deposition. Sci. Total Environ..

[4] Mahapatra B., Dhal N.K., Dash A.K., Panda B.P., Panigrahi K.C.S., Pradhan A. (2019). Perspective of Mitigating Atmospheric Heavy Metal Pollution: Using Mosses as Biomonitoring and Indicator Organism. Environ. Sci. Pollut. Res..

[5] Di Palma A., Capozzi F., Spagnuolo V., Giordano S., Adamo P. (2017). Atmospheric Particulate Matter Intercepted by Moss-Bags: Relations to Moss Trace Element Uptake and Land Use. Chemosphere.

[6] Lazo P., Kika A., Qarri F., Bekteshi L., Allajbeu S., Stafilov T. (2022). Air Quality Assessment by Moss Biomonitoring and Trace Metals Atmospheric Deposition. Aerosol Air Qual. Res..

[7] Zhou X., Chen Q., Liu C., Fang Y. (2017). Using Moss to Assess Airborne Heavy Metal Pollution in Taizhou, China. Int. J. Environ. Res. Public Health.

[8] Cowden P., Aherne J. (2019). Assessment of Atmospheric Metal Deposition by Moss Biomonitoring in a Region under the Influence of a Long Standing Active Aluminium Smelter. Atmos. Environ..

[9] Gatziolis D., Jovan S., Donovan G., Amacher M., Monleon V. (2016). Elemental Atmospheric Pollution Assessment via Moss-Based Measurements in Portland, Oregon.

[10] Mentese S., Yayintas Ö.T., Bas B., İrkin L.C., Yilmaz S. (2021). Heavy Metal and Mineral Composition of Soil, Atmospheric Deposition, and Mosses with Regard to Integrated Pollution Assessment Approach. Environ. Manag..

[11] Zheng G., Gu J., Zhao W., Zhang Y., Guan Z., Lei M., He C. (2024). Spatial, Geographical, Climatic, and Edaphic Influences on Moss Community Structure: A Case Study from Qinhuangdao, China. Forests.

[12] De Agostini A., Cortis P., Cogoni A. (2020). Monitoring of Air Pollution by Moss Bags around an Oil Refinery: A Critical Evaluation over 16 Years. Atmosphere.

[13] Nickel S., Schröder W. (2019). Correlating Elements Content in Mosses Collected in 2015 across Germany with Spatially Associated Characteristics of Sampling Sites and Their Surroundings. Environ. Sci. Eur..

[14] Aničić M., Tomašević M., Tasić M., Rajšić S., Popović A., Frontasyeva M.V., Lierhagen S., Steinnes E. (2009). Monitoring of Trace Element Atmospheric Deposition Using Dry and Wet Moss Bags: Accumulation Capacity versus Exposure Time. J. Hazard. Mater..

[15] Stafilov T., Bačeva Andonovska K., Šajn R., Jeftimova M. (2025). Assessing the Distribution of Potentially Toxic Elements in Bryophytes in Relation to Surface Soil Contamination in the Veles Region, North Macedonia. Plants.

[16] Świsłowski P., Nowak A., Wacławek S., Silvestri D., Rajfur M. (2022). Bioaccumulation of Trace Elements from Aqueous Solutions by Selected Terrestrial Moss Species. Biology.

[17] Wang X., Yuan W., Feng X., Wang D., Luo J. (2019). Moss Facilitating Mercury, Lead and Cadmium Enhanced Accumulation in Organic Soils over Glacial Erratic at Mt. Gongga, China. Environ. Pollut..

[18] Radziemska M., Mazur Z., Bes A., Majewski G., Gusiatin Z.M., Brtnicky M. (2019). Using Mosses as Bioindicators of Potentially Toxic Element Contamination in Ecologically Valuable Areas Located in the Vicinity of a Road: A Case Study. Int. J. Environ. Res. Public Health.

[19] Zhou X., Li J., Yan P., Lu N., Lu L., Ni Q., Zhang J., Fang Y. (2025). Mosses as Biomonitors of Atmospheric Trace Metal and Nitrogen Deposition: Spatial Distribution and Temporal Trend in Yancheng, China. Plants.

[20] Kempter H., Krachler M., Shotyk W., Zaccone C. (2017). Validating Modelled Data on Major and Trace Element Deposition in Southern Germany Using Sphagnum Moss. Atmos. Environ..

[21] Urošević M.A., Krmar M., Radnović D., Jovanović G., Jakšić T., Vasić P., Popović A. (2020). The Use of Moss as an Indicator of Rare Earth Element Deposition over Large Area. Ecol. Indic..

[22] Schröder W., Nickel S. (2019). Moss Species-Specific Accumulation of Atmospheric Deposition?. Environ. Sci. Eur..

[23] Zhang L., Cheng P., Hou X., Wang D., Liu X., Liu Q., Dong G., Zhou J., Jiang H., Tang L. (2024). Long-Range Transboundary Transport of Iodine-129 from South Asia to the Southern Tibetan Plateau Revealed by Moss and Lichen. Environ. Sci. Technol. Lett..

[24] Yurukova L., Tsakiri E., Cayir A. (2009). Cross-Border Response of Moss, Hypnum Cupressiforme Hedw., to Atmospheric Deposition in Southern Bulgaria and Northeastern Greece. Bull. Environ. Contam. Toxicol..

[25] Coşkun M., Yurukova L., Çayir A., Coşkun M., Gecheva G. (2009). Cross-Border Response of Mosses to Heavy Metal Atmospheric Deposition in Southeastern Bulgaria and European Turkey. Environ. Monit. Assess..

[26] Stebel K., Christensen G., Derome J., Grekelä I. (2007). State of the Environment in the Norwegian, Finnish and Russian Border Area. Finn. Environ..

[27] Betsou C., Tsakiri E., Kazakis N., Vasilev A., Frontasyeva M., Ioannidou A. (2019). Atmospheric Deposition of Trace Elements in Greece Using Moss Hypnum Cupressiforme Hedw. as Biomonitors. J. Radioanal. Nucl. Chem..

[28] Zinicovscaia I., Hramco C., Duliu O.G., Vergel K., Culicov O.A., Frontasyeva M.V., Duca G. (2017). Air Pollution Study in the Republic of Moldova Using Moss Biomonitoring Technique. Bull. Environ. Contam. Toxicol..

[29] Chaligava O., Shetekauri S., Badawy W.M., Frontasyeva M.V., Zinicovscaia I., Shetekauri T., Kvlividze A., Vergel K., Yushin N. (2021). Characterization of Trace Elements in Atmospheric Deposition Studied by Moss Biomonitoring in Georgia. Arch. Environ. Contam. Toxicol..

[30] Frontasyeva M., Harmens H., Uzhinskiy A., Chaligava O. (2020). Mosses as Biomonitors of Air Pollution: 2015/2016 Survey on Heavy Metals, Nitrogen and POPs in Europe and Beyond. Report of the ICP Vegetation Moss Survey Coordination Centre.

[31] Oishi Y. (2019). Moss as an Indicator of Transboundary Atmospheric Nitrogen Pollution in an Alpine Ecosystem. Atmos. Environ..

[32] Zinicovscaia I., Chaligava O., Yushin N., Grozdov D., Vergel K., Hramco C. (2022). Moss Biomonitoring of Atmospheric Trace Element Pollution in the Republic of Moldova. Arch. Environ. Contam. Toxicol..

[33] Aboal J.R., Fernández J.A., Boquete T., Carballeira A. (2010). Is It Possible to Estimate Atmospheric Deposition of Heavy Metals by Analysis of Terrestrial Mosses?. Sci. Total Environ..

[34] Bejan I., Sochircă V., Nagacevschi T., Ţîţu P. (2022). Spatial Study of Soil Erosion in the Republic of Moldova. Present Environ. Sustain. Dev..

[35] Chaligava O., Zinicovscaia I., Peshkova A., Yushin N., Frontasyeva M., Vergel K., Nurkassimova M., Cepoi L. (2024). Major and Trace Airborne Elements and Ecological Risk Assessment: Georgia Moss Survey 2019–2023. Plants.

[36] Zinicovscaia I., Hramco C., Chaligava O., Yushin N., Grozdov D., Vergel K., Duca G. (2021). Accumulation of Potentially Toxic Elements in Mosses Collected in the Republic of Moldova. Plants.

[37] Kabata-Pendias A., Szteke B. (2015). Trace Elements in Abiotic and Biotic Environments.

[38] Tarita A., Braşoveanu V., Jigău G. (2021). The Content of Heavy Metals in the Soils of the State Protected Natural Areas in the South-Eastern Area of the Republic of Moldova. Lucrări Ştiinţifice. Seria Horticultură.

[39] Gambashidze G.O., Urushadze T.F., Blum W.E., Mentler A.F. (2014). Heavy Metals in Some Soils of Western Georgia. Eurasian Soil. Science.

[40] UNECE (2014). Environmental Performance Reviews: Republic of Moldova: 3rd Review.

[41] UNECE (2016). Environmental Performance Reviews: Georgia: 3rd Review.

[42] Aničić Urošević M., Ilić M., Radnović D., Vergel K., Yushin N., Chaligava O., Zinicovscaia I. (2024). Comparative Biomonitoring of Airborne Potentially Toxic Elements Using Mosses (*Hypnum cupressiforme*, *Brachythecium* spp.) and Lichen (*Evernia prunastri*) over Remote Areas. Environ. Sci. Pollut. Res..

[43] Capozzi F., Di Palma A., Sorrentino M.C., Adamo P., Giordano S., Spagnuolo V. (2020). Morphological Traits Influence the Uptake Ability of Priority Pollutant Elements by Hypnum Cupressiforme and Robinia Pseudoacacia Leaves. Atmosphere.

[44] Renaudin M., Leblond S., Meyer C., Rose C., Lequy E. (2018). The Coastal Environment Affects Lead and Sodium Uptake by the Moss Hypnum Cupressiforme Used as an Air Pollution Biomonitor. Chemosphere.

[45] Beck H.E., McVicar T.R., Vergopolan N., Berg A., Lutsko N.J., Dufour A., Zeng Z., Jiang X., van Dijk A.I.J.M., Miralles D.G. (2023). High-Resolution (1 Km) Köppen-Geiger Maps for 1901–2099 Based on Constrained CMIP6 Projections. Sci. Data.

[46] World Meteorological Organization (2017). WMO Guidelines on the Calculation of Climate Normals.

[47] Pesaresi M., Schiavina M., Politis P., Freire S., Krasnodębska K., Uhl J.H., Carioli A., Corbane C., Dijkstra L., Florio P. (2024). Advances on the Global Human Settlement Layer by Joint Assessment of Earth Observation and Population Survey Data. Int. J. Digit. Earth.

[48] Yan G., Tang G., Lu D., Ma J., Yang X., Li F. (2024). Distinguishing the Intervalley Plain from the Intermountain Flat for Landform Mapping Using the Sightline Algorithm. ISPRS Int. J. Geoinf..

[49] Woods T., McKeen T., Cunningham A., Priyatikanto R., Sorichetta A., Tatem A., Bondarenko M. (2025). WorldPop High Resolution, Harmonised Annual Global Geospatial Covariates. https://eprints.soton.ac.uk/500663/.

[50] Yamazaki D., Ikeshima D., Neal J.C., O’Loughlin F., Sampson C.C., Kanae S., Bates P.D. MERIT DEM: A New High-Accuracy Global Digital Elevation Model and Its Merit to Global Hydrodynamic Modeling. Proceedings of the AGU Fall Meeting.

[51] Abatzoglou J.T., Dobrowski S.Z., Parks S.A., Hegewisch K.C. (2018). TerraClimate, a High-Resolution Global Dataset of Monthly Climate and Climatic Water Balance from 1958–2015. Sci. Data.

[52] Patil I. (2021). Visualizations with Statistical Details: The’ggstatsplot’approach. J. Open Source Softw..

[53] Lê S., Josse J., Husson F. (2008). FactoMineR: An R Package for Multivariate Analysis. J. Stat. Softw..

[54] Kassambara A., Mundt F. (2020). factoextra: Extract and Visualize the Results of Multivariate Data Analyses. https://CRAN.R-project.org/package=factoextra.

