# Supplementary material for: Environmental Drivers of Trace Element Variability in Hypnum cupressiforme Hedw.: A Cross-Regional Moss Biomonitoring Study in Georgia and the Republic of Moldova
Source: Plants (Basel). 2025 Jul 3;14(13):2040. doi: 10.3390/plants14132040 (PMC12252356; doi:10.3390/plants14132040)
Supplement: Supplementary file 1 [file plants-14-02040-s001.zip › plants-3689722-supplementary.pdf]

Table S1. Quality control of measurements and LOD values for the determined elements.

<table>
<tr><th rowspan="2"></th><th rowspan="2">Elements</th><th colspan="3">M2</th><th colspan="3">OBTL-5</th><th rowspan="2">LOD, mg/kg</th></tr>
<tr><th>Experimental value, mg/kg</th><th>Certified value, mg/kg</th><th>Recovery, %</th><th>Experimental value, mg/kg</th><th>Certified value, mg/kg</th><th>Recovery, %</th></tr>
<tr><th rowspan="13">ICP-OES</th><td>Al</td><td>163.51</td><td>175</td><td>93</td><td>1840.8</td><td>1981</td><td>93</td><td>0.4965</td></tr>
<tr><td>Ba</td><td>17.23</td><td>17.6</td><td>98</td><td>64.8</td><td>67.4</td><td>96</td><td>0.0041</td></tr>
<tr><td>Cd</td><td>0.45</td><td>0.454</td><td>99</td><td>2.3</td><td>2.64</td><td>87</td><td>0.0001</td></tr>
<tr><td>Co</td><td>0.89</td><td>0.9</td><td>99</td><td>0.9</td><td>0.98</td><td>92</td><td>0.0004</td></tr>
<tr><td>Cr</td><td>0.93</td><td>0.92</td><td>101</td><td>-</td><td>-</td><td>-</td><td>0.0001</td></tr>
<tr><td>Cu</td><td>66.75</td><td>68.1</td><td>98</td><td>9.6</td><td>10.1</td><td>95</td><td>0.0005</td></tr>
<tr><td>Fe</td><td>255.95</td><td>245</td><td>104</td><td>1496.1</td><td>1491</td><td>100</td><td>1.024</td></tr>
<tr><td>Mn</td><td>322.03</td><td>357</td><td>90</td><td>161.5</td><td>180</td><td>90</td><td>0.1136</td></tr>
<tr><td>Ni</td><td>14.11</td><td>14.8</td><td>95</td><td>6.7</td><td>8.5</td><td>79</td><td>0.0001</td></tr>
<tr><td>Pb</td><td>6.56</td><td>5.86</td><td>112</td><td>1.8</td><td>2.01</td><td>90</td><td>0.001</td></tr>
<tr><td>Sr</td><td>-</td><td>-</td><td>-</td><td>97.6</td><td>105</td><td>93</td><td>0.0034</td></tr>
<tr><td>V</td><td>1.37</td><td>1.21</td><td>113</td><td>3.5</td><td>4.12</td><td>85</td><td>0.0014</td></tr>
<tr><td>Zn</td><td>35.80</td><td>35.2</td><td>102</td><td>49.8</td><td>52.4</td><td>95</td><td>0.0009</td></tr>
<tr><th rowspan="11">NAA*</th><th>Elements</th><th colspan="3">CRMs</th><th>Experimental value, mg/kg</th><th>Certified value, mg/kg</th><th>Recovery, %</th><th>LOD, mg/kg</th></tr>
<tr><td>Al</td><td colspan="3">1575a</td><td>557</td><td>580</td><td>96</td><td>3.2</td></tr>
<tr><td>Ba</td><td colspan="3">2711</td><td>725.9</td><td>726</td><td>100</td><td>1.7</td></tr>
<tr><td>Co</td><td colspan="3">1633c</td><td>121.06</td><td>120.6</td><td>100</td><td>0.03</td></tr>
<tr><td>Cr</td><td colspan="3">2709a</td><td>113</td><td>130</td><td>87</td><td>1.3</td></tr>
<tr><td>Fe</td><td colspan="3">2709a</td><td>32232</td><td>33600</td><td>96</td><td>0.9</td></tr>
<tr><td>Mn</td><td colspan="3">1547</td><td>94</td><td>98</td><td>96</td><td>0.08</td></tr>
<tr><td>Ni</td><td colspan="3">1632c</td><td>9.3</td><td>9.32</td><td>100</td><td>1.4</td></tr>
<tr><td>Sr</td><td colspan="3">2711</td><td>257</td><td>245</td><td>105</td><td>1.5</td></tr>
<tr><td>V</td><td colspan="3">2710</td><td>76</td><td>76.6</td><td>99</td><td>0.18</td></tr>
<tr><td>Zn</td><td colspan="3">1632c</td><td>10.7</td><td>12.1</td><td>88</td><td>0.4</td></tr>
<tr><th rowspan="5">AAS</th><th rowspan="2">Elements</th><th colspan="6">OBTL-5</th><th rowspan="2">LOD, mg/kg</th></tr>
<tr><th colspan="2">Experimental value, mg/kg</th><th colspan="2">Certified value, mg/kg</th><th colspan="2">Recovery, %</th></tr>
<tr><td>Cd</td><td colspan="2">2.6</td><td colspan="2">2.64</td><td colspan="2">98</td><td>0.0001</td></tr>
<tr><td>Cu</td><td colspan="2">9.8</td><td colspan="2">10.1</td><td colspan="2">97</td><td>0.0005</td></tr>
<tr><td>Pb</td><td colspan="2">1.94</td><td colspan="2">2.01</td><td colspan="2">97</td><td>0.001</td></tr>
</table>

* Since NAA relies on relative comparison, results from just one reference material (experimental vs. certified value) are included.

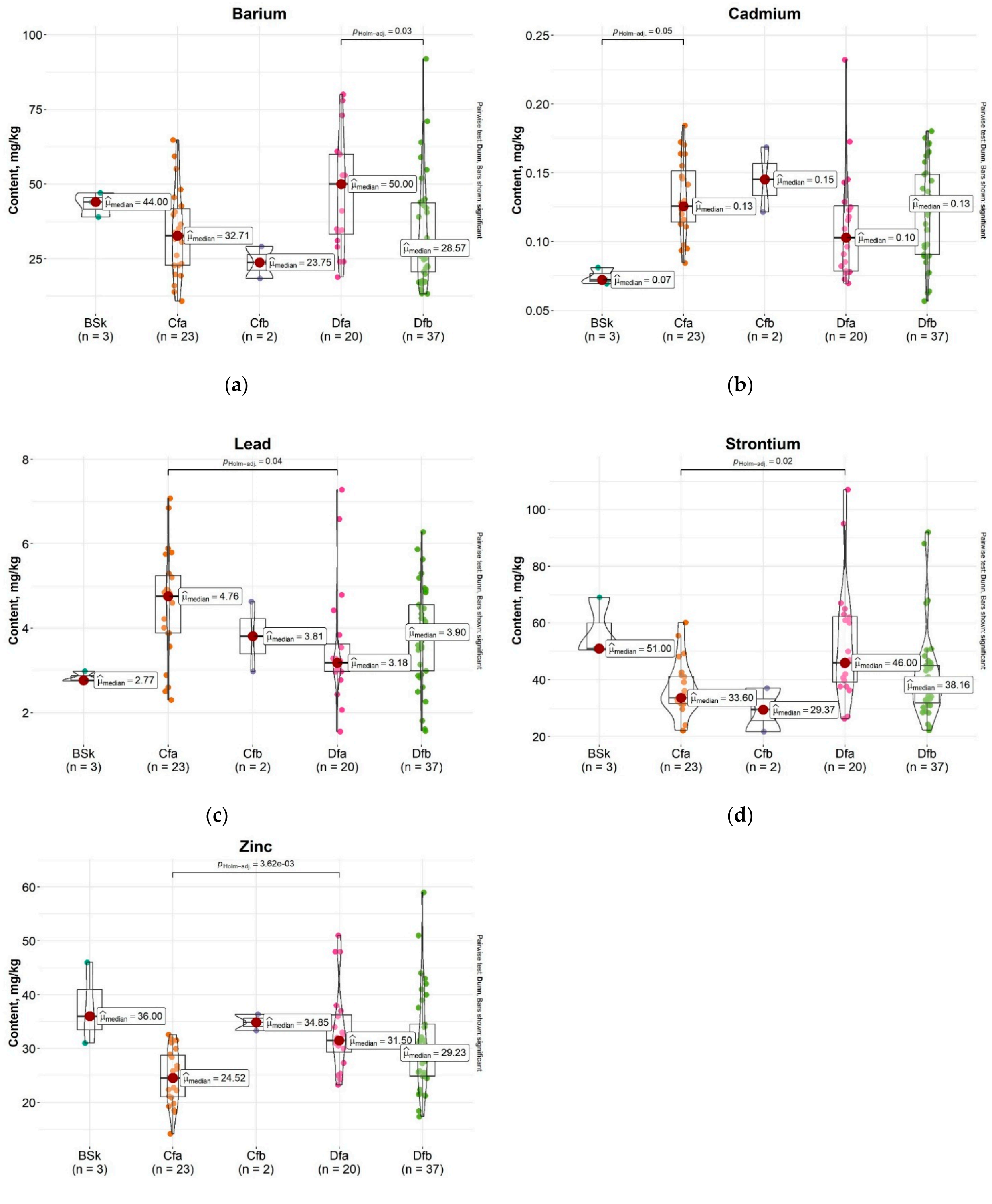

Figure S1. Boxplots showing elemental content in mosses across climate zones for: (a) Barium; (b) Cadmium; (c) Lead; (d) Strontium; (e) Zinc. Significant pairwise differences (Kruskal–Wallis with Dunn's post hoc, $p < 0.05$) are indicated with connecting lines.
